# Intensity‐modulated radiation therapy dose verification using fluence and portal imaging device

**DOI:** 10.1120/jacmp.v17i1.5899

**Published:** 2016-01-08

**Authors:** Iori Sumida, Hajime Yamaguchi, Indra J. Das, Hisao Kizaki, Keiko Aboshi, Mari Tsujii, Yuji Yamada, Osamu Suzuki, Yuji Seo, Fumiaki Isohashi, Kazuhiko Ogawa

**Affiliations:** ^1^ Department of Radiation Oncology Osaka University Graduate School of Medicine Suita Osaka Japan; ^2^ Department of Radiation Oncology NTT West Osaka Hospital Tennoji‐ku Osaka Japan; ^3^ Department of Radiation Oncology Indiana University School of Medicine Indianapolis Indiana USA

**Keywords:** IMRT, EPID, dose reconstruction, fluence‐based dose calculation, QA

## Abstract

Patient‐specific quality assurance for intensity‐modulated radiation therapy (IMRT) dose verification is essential. The aim of this study is to provide a new method based on the relative error distribution by comparing the fluence map from the treatment planning system (TPS) and the incident fluence deconvolved from the electronic portal imaging device (EPID) images. This method is validated for 10 head and neck IMRT cases. The fluence map of each beam was exported from the TPS and EPID images of the treatment beams were acquired. Measured EPID images were deconvolved to the incident fluence with proper corrections. The relative error distribution between the TPS fluence map and the incident fluence from the EPID was created. This was also created for a 2D diode array detector. The absolute point dose was measured with an ionization chamber, and the dose distribution was measured by a radiochromic film. In three cases, MLC leaf positions were intentionally changed to create the dose error as much as 5% against the planned dose and our fluence‐based method was tested using gamma index. Absolute errors between the predicted dose of 2D diode detector and of our method and measurements were 1.26%±0.65% and 0.78%±0.81% respectively. The gamma passing rate (3% global / 3 mm) of the TPS was higher than that of the 2D diode detector (p<0.02), and lower than that of the EPID (p<0.04). The gamma passing rate (2% global / 2 mm) of the TPS was higher than that of the 2D diode detector, while the gamma passing rate of the TPS was lower than that of EPID (p<0.02). For three modified plans, the predicted dose errors against the measured dose were 1.10%, 2.14%, and −0.87%. The predicted dose distributions from the EPID were well matched to the measurements. Our fluence‐based method provides very accurate dosimetry for IMRT patients. The method is simple and can be adapted to any clinic for complex cases.

PACS numbers: 87.55.D‐, 87.55.km, 87.55.Qr, 87.57.uq

## I. INTRODUCTION

The applications of intensity‐modulated radiation therapy (IMRT) have progressed to various sites in the body to provide adequate doses to targets while sparing normal tissues. To accurately deliver steep dose distributions to the patient, image‐guided devices such as electronic portal imaging devices (EPIDs), computed tomography (CT) on‐rail, infrared markers, and ultrasound systems have been used before or during irradiation. Although an EPID is commonly used during imaging to detect anatomical and field locations while conducting patient setup, these devices have also been used for dose verification with suitable algorithms and corrections.^1^, ^2^, ^3^, ^4^, ^5^, ^6^, ^7^, ^8^, ^9^, ^10^, ^11^, ^12^, ^13^, ^14^, ^15^, ^16^, ^17^, ^18^, ^19^, ^20^ Various groups have used EPID image conversion via deconvolution of the incident fluence distribution and optical glare kernel to dose distribution via the convolution kernel.^4^, ^6^, ^8^, ^9^ These approaches have provided efficient IMRT dose verification for patient‐specific quality assurance (QA). IMRT QA is commonly verified in each patient using a two‐dimensional (2D) diode or chamber array detector. However, Nelms et al.^(21)^ demonstrated a poor correlation between the planar IMRT QA passing rates and clinically relevant patient dose errors, which may occur in the predicted dose distributions involving only three dimensions (3D). Kruse^(22)^ also reported insensitivity between the per‐beam QA gamma passing rate and dosimetric accuracy in 3D. These studies suggest the importance of not only evaluating dose verification in 3D over a clinically relevant anatomic field but also of predicting dose distributions on the basis of QA results,^23^, ^24^ or measurement‐guided dose reconstruction (MGDR).^(25)^ The measured data are usually derived from 2D or 3D detector arrays and include EPID dosimetry.^(26)^ A few studies have used measured fluence data^27^, ^28^ and required dose calculation engines, such as pencil beam convolution algorithms, to reconstruct the 3D dose distributions.

In our previous study, the relative local error distribution in 2D was created as a per‐beam QA for IMRT, after which the error distribution was forward‐projected to the dose grid data of each beam to create QA results‐based predicted dose distributions where prediction algorithms had been validated.^(24)^ Assuming that the relative error distribution determined in the phantom measurement would be maintained in air measurements and that the hypothesis is correct, it would likely be more efficient to perform per‐beam QA under fluence verification in air without a dose comparison; in other words, to predict dose distribution on the basis of the MGDR. The aim of this study was to validate this air fluence approach for predicting IMRT dose distribution in 3D using EPID.

## II. MATERIALS AND METHODS

**Figure 1:**
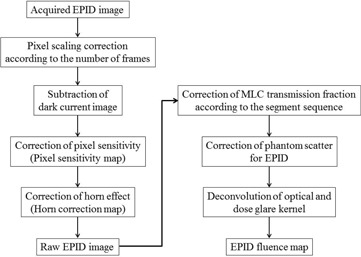
Conversion process from the EPID image to EPID fluence.

A linear accelerator with a 6 MV X‐ray beam and dose rate of 300 monitor units (MU) per min (ONCOR Impression PLUS; Siemens Medical Systems, Concord, CA) was used. An EPID (OPTIVUE 1000ST; Siemens Medical Systems) was used to acquire images comprising 1024×1024 matrices, with a detector size of $0.4\times 0.4\;{\text{mm}}^{2}$. The source‐imager distance (SID) was set to 130 cm for all image acquisitions. A XiO treatment planning system (Elekta Instrument AB, Stockholm, Sweden) was used to calculate dose distributions and export a fluence map of each beam. The dose calculation and fluence map resolutions were $1\times 1\times 1\;{\text{mm}}^{3}$ and $1\times 1\;{\text{mm}}^{2}$, respectively. The process used to convert an EPID image to incident fluence is shown in Fig. 1. Several necessary corrections are presented in detail in the following sections.

### A. Correction of EPID response against the fluence map for an open field

At 130 cm, the possible image acquisition area is $315\times 315\;{\text{mm}}^{2}$ at the isocenter plane. Because no additional build‐up material is placed on the EPID, the horn effect, which derives from a flattening filter as well as the fluence map of the TPS in air, may be visible in an EPID image. The fluence map was defined at the isocenter plane, and the acquired EPID image was scaled from 130 cm to a source‐axis distance of 100 cm, resulting in an EPID‐derived pixel resolution of 0.31 mm at the isocenter plane. A fluence map of the $30\times 30\;{\text{mm}}^{2}$ open field was calculated in the TPS at an irradiation dose of 10 MU to the EPID. This was selected because the OPTIVUE 1000ST experiences pixel variations and reduced reproducibility at doses <10 MU.^18^, ^20^ Regarding the hardware correction the EPID image at 10 MU was used to correct for the combination of pixel sensitivity variations^(7)^ and the horn effect^4^, ^8^ because the pixel profile created by an automatic flood‐field correction in the image acquisition software was almost flat (data not shown). Despite the flat pixel profile, the 2D pixel sensitivity map (PSM) was calculated as the ratio of the off‐axis pixel value to the central axis, as discussed by Greer.^(7)^ After creating the PSM, a raw EPID image was calculated by multiplying the acquired image by the number of frames,^(20)^ subtracting the dark current image, and dividing by the PSM. Regarding the TPS correction to correct the horn effect on the fluence map, both the fluence map and raw $30\times 30\;{\text{mm}}^{2}$ EPID image were normalized to the central axis value, after which a horn correction map (HCM) was calculated by dividing the normalized raw EPID data by the normalized fluence map. Both PSM and HCM were used to correct the horn effect in hardware and software approaches. The correction is described by the following equation:

(1) $$P_{raw}(i,j)=\frac{\left\{(\sum_{a=1}^{n}P_{a}(i,j)\times N_{a})-P_{dark}(i,j)\right\}}{PSM\cdot HCM}$$

where $P_{raw}(i,j)$ denotes a raw pixel value at location *i, j* on the EPID, $P_{a}(i,j)$ denotes a pixel value on an acquired image for the *ath* segment, $N_{a}$ denotes the number of frames for the *ath* segment, *n* denotes the number of segments, and $P_{dark}(i,j)$ denotes a pixel value in the current dark image.

### B. MLC transmission fraction factor

A step‐and‐shoot IMRT technique was used in this study, therefore requiring that the MLC‐transmitted radiation, as well as the incident beam, should be accounted for in the irradiated EPID image. The OPTIVUE 1000ST comprises amorphous, indirect‐type silicon detectors; therefore, the gadolinium oxysulfide phosphor layer, which has a high atomic number, over‐responds to low‐energy X‐rays transmitted from the MLC at off‐axis positions relative to the central axis position and according to the presence of a flattening filter, as shown by Vial et al.^(14)^ According to this study, the MLC transmission fraction (MTF) factor (MTFF), a function of the central off‐axis distance via leaf movement, can be calculated from the two irradiated EPID images as an open field of $30\times 30\;{\text{cm}}^{2}$, and a closed field by MLC leaves. The MTFF is assumed to be radially symmetrical. The off‐axis distance was up to 12 cm on one side because the beam flat region is 80% of a 30 cm wide field. The fraction value was defined as the fraction of MU irradiation from the closed field against the total MU irradiation from the closed and open fields. For example, a fraction value of 0.1 indicates a 10 MU closed field and 90 MU open field. The fraction values ranged between 0.1 and 0.9 at intervals of 0.1.

According to each segment shape in each beam derived from the DICOM RT‐plan file, the MTF factor MTFF(i, j) at each pixel location i, j on an EPID image was calculated using a look‐up table with 2D interpolation, as functions of the fraction value and the distance from the beam central axis. At a fraction value of <0.1, the MTFF(i, j) at a fraction value of 0.1 was used. At a fraction value of >0.9, the MTFF(i, j) at a fraction value of 0.9 was used. If the off‐axis distance on an EPID exceeded 12 cm, the MTFF(i, j) was set to zero, indicating no transmission of radiation to the EPID via the MLC. Raw pixel values were corrected using the MTFF(i, j) as described in the following equation:

(2) $$P_{MTF}(i,j)=P_{raw}(i,j)\times (1+MTFF(i,j))$$

where $P_{\text{MTF}}\;(i,\;j)$ denotes a pixel value corrected using the *MTFF* (*i, j*).

### C. Phantom scatter correction on an EPID

The EPID is assumed to comprise high‐density scattering material; therefore, a reference phantom scatter ratio against a $10\times 10\;{\text{cm}}^{2}$ open field is likely necessary for comparisons between a converted incident fluence on EPID in air and fluence maps from the TPS. First, the total scatter factor ($S_{\text{cp}}$) was measured in square fields ranging from $2\times 2\;{\text{cm}}^{2}$ to $20\times 20\;{\text{cm}}^{2}$ on EPID at doses of 100 MU each. The mean pixel values in the 10 pixel×10 pixel region of interest were calculated in each field and normalized to the values in a reference field ($10\times 10\;{\text{cm}}^{2}$). The collimator scatter factors ($S_{c}$), also at field sizes of $2\times 2\;{\text{cm}}^{2}$ to $20\times 20\;{\text{cm}}^{2}$, were exported from the TPS. On EPID, the phantom scatter factor ($S_{p}$) was calculated as the $S_{\text{cp}}$ divided by the $S_{c}$. To access the $S_{p}$ at different equivalent field sizes, a second‐order polynomial curve fit was used. A DICOM RT‐plan file was used to calculate the equivalent square field size of each segment in IMRT. The equivalent field size was calculated as the square root of the area that comprised the MLC leaf opening of each segment. On raw EPID images, the pixel value belonging to each segment was corrected according to the Sp of the corresponding segment, as shown in Eq. (3):

(3) $$P_{Sc}(i,j)=\frac{P_{MTF}(i,j)}{S_{p,k}}$$

where $P_{\text{Sc}}(i,\;j)$ denotes the pixel value after phantom scatter correction on an EPID, $P_{\text{MTF}}(i,\;j)$ denotes the corrected pixel value calculated from Eq. (2), and $S_{p,k}$ denotes the phantom scatter factor for the kth segment.

### D. Deconvolution of the dose glare kernel on EPID

As the OPTIVUE 1000ST is an indirect‐type flat panel detector, incident photon irradiation of the EPID generates optical scatter at the amorphous silicon layer and generates dose deposition.^(6)^ To convert the EPID image to the incident fluence in air, both optical and phantom scatter must be removed. The measured EPID image, corrected as described above, was deconvolved to the incident fluence according to the deconvolution of the combined optical and dose glare kernel, as proposed by Warkentin et al.^(4)^ The optical and dose glare kernel, $K_{\text{glare}}(r)$, as a function of the radial distance r cm from the beam central axis, is used in the following equation:

(4) $$K_{glare}(r)=e^{-C_{1}r}+C_{2}e^{-C_{3}r}$$

The parameters $C_{1},\;C_{2}$, and $C_{3}$ are empirically derived fitting coefficients between the fluence map and raw EPID image at a $10\times 10\;{\text{cm}}^{2}$ open field. These values were $30\;{\text{cm}}^{-1},\;0.046$, and, $2.7\;{\text{cm}}^{-1}$ for $C_{1},\;C_{2}$, and $C_{3}$, respectively. Using Eq. (4), the $P_{\text{Sc}}i,\;j$) was deconvolved by the following equation:

(5) $$P_{EPID\;fluence}(i,j)=P_{Sc}(i,j)⊗K_{glare}(r)$$

where $P_{\text{EPID fluence}}(i,\;j)$ denotes the pixel value after the deconvolution of $K_{\text{glare}}(r)$, which results in an incident fluence.

### E. Pixel value to MU conversion

Renner et al.^(6)^ described a method for pixel correction. Briefly, the corrected pixel value is converted to a relative monitor unit (RMU) using a calibration curve generated between the pixel value and MU. The TPS fluence map provides a relative number with respect to a collimator scatter factor of 1.0 in a $10\times 10\;{\text{cm}}^{2}$ field; therefore, the calibration curve was measured on an EPID on the same field size, with MUs ranging from 10 to 100 MU. The curve fitting function was created from the correlation between the corrected pixel value and MU and was used to convert corrected pixel values to the RMUs.

### F. Predicted dose reconstruction from the measured data

Two predicted dose patterns were reconstructed from the measured QA data. One pattern was based on a fluence comparison between the TPS fluence map and measured corrected EPID fluence. The other pattern was based on a dose comparison between the calculated TPS dose distribution and the measured 2D diode array dose distribution (MapCHECK; Sun Nuclear Corporation, Melbourne, FL). The details of our predicted dose reconstruction method have been reported elsewhere.^(24)^ The dose reconstruction process in this study differs from that of the previous study with respect to the error map creation step. To reiterate, a fluence comparison, rather than a dose comparison, was performed to create the error map. However, a dose comparison was also performed as a reference. The relative error map was assumed to remain the same between the fluence comparison and dose comparison during comparisons of calculated and measured values (i.e., fluence in air or doses in a water equivalent phantom).

Ten head and neck cancer patients who underwent IMRT via fixed gantry step‐and‐shoot delivery were selected for this study. First, each treatment plan (2 Gy/fraction) was developed; for cases in which the MU of a specified segment received less than 10 MU, the prescribed dose of 2 Gy was rescaled to each segment MU exceeding 10 MU because of the unstable linearity and repeatability of EPID.^18^, ^20^ For fluence comparison, the fluence map for each beam was exported from the TPS, and the EPID image for each beam was acquired using the same MU and gantry angle as the treatment plan. To correct for EPID sag and translation at the treatment gantry angle with respect to the beam central axis, a reticle plate, in which two orthogonal tungsten wires were matched to the beam central axis, was inserted to the shadow tray followed by irradiation to the EPID. The sag and translation offset value correction for the EPID was recorded and used to match the EPID image to the beam central axis.^(29)^ The measured EPID image for each beam was converted to an image using (1), (5), followed by pixel‐to‐MU conversion.

To create an error map between the TPS and MU‐converted EPID fluence, the fluence map location with the maximum fluence value was used as a normalization point from which to scale the EPID fluence map according to the method described by Partridge et al.,^(30)^ as a comparison between the fluence map and EPID fluence in air. In contrast, to create an error map between the TPS dose plane and the measured dose in each beam, a 2D diode array was placed on the treatment couch, followed by beam irradiation at gantry 0. The source‐to‐detector distance was set at 100 cm, and the depth was set at 5 cm. The calculated dose distribution in the coronal plane at a depth of 5 cm was exported from the TPS and compared with the measured data to create an error map. The resolution of both the fluence map and EPID fluence was $1\times 1\;{\text{mm}}^{2}$ as a result of resampling with a pixel interval of 0.31 mm at the isocenter plane. Using the commercial software interpolation function, the resolutions of the calculated dose plane and 2D diode array were $1\times 1\;{\text{mm}}^{2}$ and $5\times 5\;{\text{mm}}^{2}$, respectively. For each beam, the error maps for the fluence comparison and dose comparison were incorporated into the 3D dose grid, which had been exported from the TPS in DICOM RT‐dose format, to create the predicted dose distribution.

### G. Validation of the predicted dose and dose distribution

Predicted dose validation was performed in ten head and neck cancer patients. Absolute dose measurements and dose distributions in the axial plane were verified using an ionization chamber and EBT‐film, respectively. A water‐equivalent phantom and ionization chamber (PTW PinPoint 31016 chamber; PTW, Freiburg, Germany) were used to measure absolute doses. A verification phantom (I'm*RT* phantom; IBA Dosimetry GmbH, Schwarzenbruck, Germany) and radiochromic film (EBT2; Ashland Specialty Ingredients, Wayne, NJ) inserted in the isocenter plane were used for film verification. One location in the high‐dose and low‐dose gradient regions was selected for dose measurement.

For three patients, the MLC leaf positions were manually modified to create plans with dose errors. Other machine parameters (e.g., gantry angle, collimator angle, and MU in each segment) remained the same as in the original plan. The predicted doses reconstructed from the EPID measurement‐based and 2D diode array measurement‐based error maps were compared with the measured data; the absolute dose and dose distribution as measured using film were used as references. Three film verification evaluations were performed: relative dose difference, distance to agreement (DTA), and gamma evaluation pass rate (criteria: 3% global / 3 mm, 2% global / 2 mm with a lower threshold value of 10%).^(31)^ A paired *t*‐test was used for comparison. Statistical significance was set at a 5% level.

## III. RESULTS

**Figure 2:**
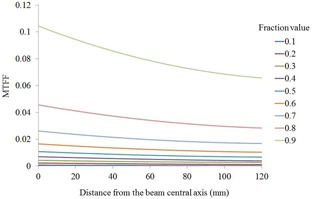
MLC transmission fraction factor (MTFF) as a function of the distance from the beam central axis.

**Figure 3:**
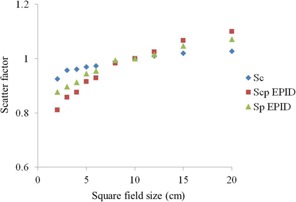
The scatter factors $S_{c},\;S_{\text{cp}}$, and $S_{p}$ as functions of the square fields derived from the EPID.

**Figure 4:**
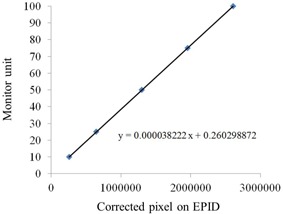
Calibration curve for OPTIVUE 1000ST. Corrected pixels were plotted against monitor units (MUs). A linear fitting formula was used for the conversion from pixel values to MUs.

MTFF(i, j), as a function of the distance from the beam central axis, is shown in Fig. 2. Each curve was fitted via polynomial regression. This factor increases according to an increase in the fraction value. The collimator scatter factor ($S_{c}$), total scatter factor ($S_{\text{cp}}$) on an EPID, and phantom scatter factor ($S_{p}$) on an EPID are shown in Fig. 3. Figure 4 presents MUs versus corrected pixels on an EPID curve. Linearity was observed with a good correlation coefficient of 0.9999.

**Figure 5:**
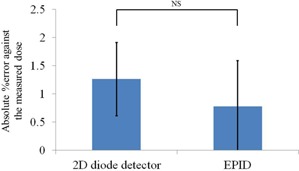
Absolute percent error against the measured dose for the 2D diode detector and EPID. Error bars represent 1 SD. There was no significant (NS) difference.

The predicted doses derived from the 2D diode detector dose comparison and EPID fluence comparison were calculated and compared with the doses measured in an ionization chamber. The absolute errors for both prediction methods in the 10 study cases are presented in Fig. 5; these errors were 1.26±0.65% and 0.78±0.81% for the dose and fluence comparisons, respectively. Although the fluence comparison had a smaller error than the dose comparison, this difference was not significant.

**Figure 6:**
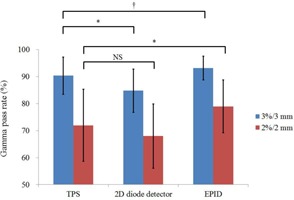
Comparisons of the gamma passing rates (criteria: 3% global / 3 mm, 2% global / 2 mm) for the calculated dose distribution of the TPS and predicted dose distributions of the 2D diode detector and EPID, compared with the film measurement. Each bar indicates the mean gamma passing rate; error bars represent 1 SD. *=p<0.02, †=p<0.04, NS=not significant.

The predicted dose distributions derived from the 2D diode detector dose comparison and EPID fluence comparison were calculated and compared with the dose distribution measured on film. As a reference, the TPS dose distribution was also compared with the measured dose distribution. As shown in Fig. 6, the gamma passing rates at the criteria of 3% global / 3 mm were 90.35%±6.88%, 84.78%±8.02%, and 93.15%±4.36% for the TPS, 2D diode detector, and EPID, respectively. The TPS gamma passing rate was higher than that of the 2D diode detector (p<0.02) but lower than that of the EPID (p<0.04). For the criteria of 2% global / 2 mm, the gamma passing rates were 71.93%±13.35%, 67.97%±11.92%, and 78.94%±9.77% for the TPS, 2D diode detector, and EPID, respectively. Although the TPS gamma passing rate was higher than that of the 2D diode detector, this difference was not significant. In contrast, the TPS gamma passing rate was lower than the EPID rate (p<0.02).

The original planned doses without leaf position modifications, modified planned doses with altered leaf positions, predicted doses, and measured doses for three cases are shown in Table 1. By intentionally changing the MLC leaf positions of three cases, the modified planned doses differed from the original planned doses by as much as −6.47%, −7.07%, and −8.03%. The predicted dose errors against the measured doses were 1.10%, 2.14%, and −0.87%. As the respective measured dose errors against the modified planned doses were −1.66%, −0.24%, and −0.57%, the respective predicted dose errors against the modified planned doses were −0.58%, 1.90%, and −1.44%.

**Figure 7:**
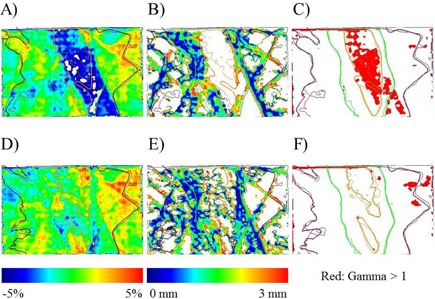
Comparisons of the dose difference (a), distance to agreement distribution (b), and gamma distribution (c) for the original planned dose vs. the measured film from one of the three modified plans. The predicted dose vs. measured data are shown in (d), (e), and (f), respectively. Gamma index values of >1 are shown in red.

Regarding dose distribution verification, the gamma passing rates between the original plans and film measurement from the modified plans for these three cases at the criteria of 3% global / 3 mm were 86.64%, 73.07%, and 76.50%. In contrast, the respective gamma passing rates between the predicted dose distributions and films in the same sequence were 96.88%, 97.49%, and 92.31%. Using the criteria of 2% global / 2 mm criteria, the gamma passing rates between the TPS and film were 74.39%, 55.84%, and 56.41%. In contrast, the respective gamma passing rates between the predicted dose distributions and films were 88.88%, 90.10%, and 73.16%. The predictions improved the gamma passing rates using both criteria. Representative dose differences, DTA distributions, and gamma distributions (3% global / 3 mm) in the axial plane from one of the cases are shown in Fig. 7.

Following manual leaf modification to the original plan, a dose error near −5% between the modified plan and original plan was achieved, as shown in Fig. 7(a). In contrast, the predicted dose distribution more closely matched the measured dose distribution shown in Fig. 7(d). The DTA distribution for the predicted distribution also provided a closer match, as shown in Fig. 7(e), when compared with the original distribution in Fig. 7(b). Gamma values of <1 and >1 are presented in white and red, respectively. The predicted gamma distribution was better than the original distribution. The gamma passing rates at the criteria of 3% global / 3 mm were 96.88% in Fig. 7(f) and 86.64% in Fig. 7(c).

**Table 1:** Comparisons of the predicted and measured doses.

*Case*	*Original Planned Dose (Gy)*	*Modified Planned Dose (Gy)*	*Predicted Dose (Gy)*	*Measured Dose (Gy)*	*Predicted Dose Error Against Modified Measured Dose (%)*	*Predicted Dose Error Against Planned Dose (%)*	*Measured Dose Error Against Modified Planned Dose (%)*
1	3.09	2.89	2.87	2.84	−0.58	1.10	−1.66
2	4.10	3.81	3.88	3.80	1.90	2.14	−0.24
3	3.61	3.32	3.27	3.30	−1.44	−0.87	−0.57

## IV. DISCUSSION

The relative error distribution between the fluence map calculated by the TPS and the measured fluence map deconvolved from an EPID image was used to predict doses on the basis of perbeam QA results. Although other approaches to dose prediction exist, these have incorporated 2D or 3D diode array detectors^(32)^ for dose measurement and even the fluence measurements in air,^(28)^ resulting in converted doses from dose calculations using beam data such as the tissue phantom ratio and off‐center ratio. In this study, the relative error distribution in air was assumed to be the same value as that for a phantom in the same situation when comparing calculations and measurements. In a previous study, the relative error distribution in each beam was determined from water‐equivalent material.^(24)^ Several researchers have deconvolved EPID images into primary fluence and subsequently into doses;^4^, ^6^, ^8^, ^9^ although corrections throughout the conversion process are necessary, our approach to fluence comparison is considered an intermediate state in other conversion approaches. If our hypothesis were correct, the same relative error distribution determined via fluence comparison would be obtained even in an air comparison. Therefore, the fluence comparison results for each beam were incorporated into the original dose determined from the DICOM RT‐dose data, and the predicted dose was reconstructed without dose calculation.

For MTFF(i, j) measurements, the maximum off‐axis distance from the central axis was 12 cm. The MTF fraction values ranged from 0.1 to 0.9. In all 10 cases, both the maximum upper jaw opening size and MLC leaf opening size were within 20 cm. Hence, the maximum off‐axis distance of 12 cm was satisfied by the correction. However, according to the leaf sequence in step‐and‐shoot IMRT, some pixels may have values of <0.1 or >0.9, resulting in correction errors. In particular, a fraction value of >0.9 indicates a region wherein most MLC leaves on an EPID were closed (covered) during irradiation. Such a region may correspond to areas outside of the field and above normal tissues with relatively lower dose constraints against the target (e.g., spinal cord).

When comparing predicted and measured doses, the mean difference of 0.78% for the fluence comparison was smaller than that of 1.26% for dose comparison, although this difference was not significant. Both predicted doses corresponded well to the measured dose.

Regarding dose distribution verification, the mean gamma passing rate for the EPID at criteria of 3% global / 3 mm was 93.2%. Although this value may not be high in comparison with the huge amount of film verification QA results published by Pulliam et al.,^(33)^ those authors stated that a gamma passing rate under the criteria of 5% global / 3 mm should exceed 90%. The criteria used in our study with respect to dose differences were stricter than those used in the earlier study. According to a survey of planar IMRT QA analysis by Nelms and Simon,^(34)^ a gamma passing rate under the criteria of 3% global/3 mm should generally range from 90% to 95%.

**Figure 8:**
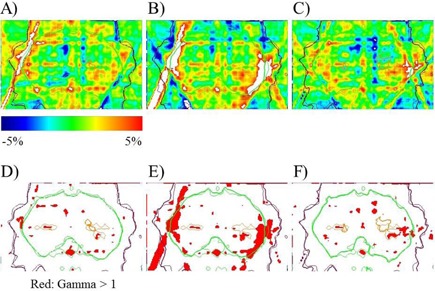
Comparisons of dose differences for the planned dose distribution (a), and the predicted dose distributions of the 2D diode detector (b) and EPID (c). Comparisons of gamma distribution are also shown in (d), (e), and (f), respectively. Gamma index values of >1 are shown in red.

In our previous study, dose prediction algorithms were validated through film verification. The gamma passing rate under the criteria of 3% global / 3 mm was 86.9%.^(24)^ Figure 8 shows the differences between the TPS dose (a) and 2D diode detector (b) and EPID predicted doses (c), when compared with film measurements. As shown in Figs. 8(a), (b), and (c), inhomogeneous distribution, possibly caused by the inherent film artifacts, was observed even in the region of homogeneous dose irradiation inside the green isodose line. Hence, the mean gamma passing rate of 93.2% was not worse than that obtained through film verification.

The mean gamma passing rates under the criteria of 3% global / 3 mm were lower for the 2D diode detector than for the TPS and EPID. In particular the rates obtained with the 2D diode detector and EPID were 84.8% and 93.2%, respectively. Despite using the same dose prediction approach, the hypothesis that the relative error maps would be the same in air and in the phantom was invalid. Differences in the gamma passing rates may have resulted from the resolution and data interpolation method associated with the 2D diode detector. The data resolutions were 1 mm, 0.31 mm, and 5 mm for the TPS, EPID, and 2D diode detector, respectively. Regarding the latter detector, the 5‐mm resolution used in this study was available in the “Dose Interpolated” data section of the MapCHECK software package. The gamma distributions under the criteria of 3% global / 3 mm are shown in Figs. 8(d), (e), and (f). The respective gamma passing rates were 96.66%, 86.40%, and 95.94%. The high‐dose regions (inside the green isodose line) of the predicted and measured doses were matched within a 5% dose difference, whereas in other regions, the gamma index exceeded 1, as shown in red along the lateral edge of the green isodose line in Fig. 8(e). These regions were derived from dose interpolation (MapCHECK software) at a detector interval of 10 mm, rather than 5 mm, at an off‐axis distance of >5 cm, which comprises a standard diode detector configuration. For head and neck IMRT, the field width along the leaf motion was relatively wider than that of prostate IMRT. Sumida et al.^(24)^ reported a dose prediction algorithm and validation for prostate IMRT. However, if this algorithm is used for relatively large targets such as head‐and‐neck and pelvic cancers, the detector resolution should be finer than the standard resolution of the MapCHECK device to compensate for the software interpolated data. A finer resolution can be achieved using the doubled detector density method, as suggested by Keeling et al.^(35)^

One limitation of this study was the requirement for MU in each segment to exceed 10 MU because the OPTIVUE 1000ST exhibits pixel variations and reduced reproducibility below MU.^18^, ^20^ Therefore, MU scaling was incorporated to solve this issue. Given the improvements in image acquisition software produced by another vendor, rather than in the EPID,^(18)^ the concept of fluence comparison adopted in this study would be expected to predict doses even for rotational IMRT. Another limitation of this study was the normalization of the incident fluence, which was deconvolved from the EPID image, to match the TPS fluence map. As the peak fluence map value depends on the MLC leaf sequence pattern and MU, if the peak value location is in a small area, a systematic error in the normalization process may occur because of the measurement accuracy of the EPID output factor in small fields. In the future, an absolute comparison will be needed to create a relative error map, thus improving the accuracy of correction to EPID.

Currently, the dose prediction approach could be used in IMRT QA to compare delivered and planned dose volume indices from dose‐volume histograms and doses. Although validation of point doses in 1D and dose distributions in 2D is possible, it would be difficult to validate dose‐volume data, as a high‐detector density is required for 3D measurements. In future, 3D dosimeters such as gel dosimeters^(36)^ and plastic scintillation detectors^(37)^ should be used to validate dose‐volume data.

When EPID‐based per‐beam QA is performed as a patient‐specific QA to predict the dose in a patient, radiation damage to the system is a concern with respect to the lifespan of the EPID. Louwe et al.^(38)^ reported that some EPIDs exhibited excellent stability for periods up to 23 months, whereas other EPIDs required more frequent replacement.

## V. CONCLUSIONS

Dose predictions were based on fluence comparisons between TPS fluence maps and incident fluence deconvolved from EPID images. For validation, the measured doses and dose distributions were compared with the corresponding predicted values. The absolute percent errors against the measured doses were <1% for fluence‐based dose predictions. To verify dose distributions, the gamma passing rates for fluence‐based values were higher than those for TPS values under the criteria of 3% global / 3 mm or 2% global / 2 mm. This dose prediction approach is, therefore, useful and efficient with respect to EPID image collection and fine spatial resolution.
